# Social media trends in obstetrics and gynecology residency programs on Instagram and X (Twitter)

**DOI:** 10.1371/journal.pone.0296930

**Published:** 2024-05-06

**Authors:** Colette M. Gnade, Chace C. Avery, Ziyi Yang, Charlotte M. Pickett, Adeoti E. Oshinowo

**Affiliations:** 1 Department of Obstetrics and Gynecology, University of Kansas, Kansas City, Kansas, United States of America; 2 Department of Obstetrics and Gynecology, University of Cincinnati, Cincinnati, Ohio, United States of America; 3 Department of Biostatistics and Health Data Science, Indiana University, Indianapolis, Indiana, United States of America; 4 Department of Obstetrics and Gynecology, University of California San Diego, La Jolla, California, United States of America; 5 Department of Obstetrics and Gynecology, Indiana University, Indianapolis, Indiana, United States of America; Saint Louis University School of Medicine, UNITED STATES

## Abstract

**Background:**

During the COVID pandemic, residency program’s social media presence increased to aid in residency recruitment by attempting to increase engagement and readily available information for applicants across specialties. However, little information exists on what characteristics and content on obstetrics and gynecology (OBGYN) residency program accounts attract more followers or engagement.

**Objectives:**

To identify social media trends in OBGYN residencies and determine which aspects of programs influence the number of followers and interaction with content posted.

**Methods:**

We performed a retrospective review of ACGME accredited OBGYN programs and determined their presence on Instagram and X in the fall of 2021. Content from the thirty programs with the most followers was analyzed independently by two authors. Multivariate analysis and a linear mixed model were used to characterize and evaluate content on Instagram and X.

**Results:**

Most programs utilized Instagram (88.5%, N = 262/296) and were managed solely by residents (84.4%, N = 108/128). Number of followers on Instagram positively correlated with features such as program size, Instagram profile duration, and Doximity rankings (p < 0.0x01). Programs on X had more followers if their profile had a longer duration, followed more individuals, or were ranked higher on Doximity. The most posted Instagram content was biographical and social in nature. Instagram posts with the highest engagement were awards and/or the Match.

**Conclusions:**

Understanding what social media content attracts more followers and increases engagement is crucial as it likely impacts OBGYN resident recruitment. Professional groups should establish guidelines for social media use in recruitment for the protection of both residents and applicants.

## Introduction

Currently there are 4.62 billion social media users making up 58.4% of the world population. This number grew by more than 10% from 2021 to 2022 [1]. This trend in social media use is paralleled in healthcare with up to 90% of workers having a social media account [2]. Increased professional content on social media has been driven by the desire to stay connected to peers in the medical community, share accurate knowledge with patients, and develop a brand [3–5]. An increase in social media influence has transformed the experience of residency trainees by impacting education, professional development, and academic scholarship [6]. The COVID pandemic has further increased residency program presence on social media as it brought significant challenges to residency recruitment, interviewing, and away rotations [5, 7–9]. The number of programs on social media is rapidly expanding but remains variable on both Instagram (40.9% neurology to 96.6% plastic surgery residency programs) and X, formerly known as Twitter, (14% of dermatology programs to 44.1% otolaryngology residency programs) [7, 10–12]. Obstetrics and gynecology (OBGYN) residency programs have increased their presence on Instagram, specifically during the pandemic [13].

Residency applicants report that a program’s social media presence influences their application, interview, and rank process. In studies with recruitment prior to 2020, around 12–29% of applicants note that social media presence influenced their recruitment [8, 14, 15]. However, after 2020, around 60%-71% of applicants reported social media impacted their perceptions of residency programs [16–18]. These studies, which are from a wide range of specialties including general surgery, anesthesia, and family medicine, have concluded that programs should consider investing resources into their social media [16–18]. Social media platforms have become, and likely will remain, an integral part of professional life and residency recruitment. However, factors to help engage applicants and create an interactive social media residency account are relatively unknown. Our study was performed to examine social media trends in OBGYN residencies, analyze program and social media characteristics associated with program followers, and analyze social media content and post engagement.

## Materials and methods

We performed a retrospective study to identify the extent of social media presence of OBGYN programs on both Instagram and X platforms as these were those most studied prior [13, 19]. Facebook was initially inquired, however, given low numbers, difficulty searching the programs, and American College of Obstetrics and Gynecology (ACOG) only utilizing Instagram and X for virtual residency showcase recruitment since 2020, this was deferred [20]. Institutional IRB exemption (#13531) was obtained for this study from Indiana University Human Research Protection Program. We complied with both Instagram and X terms of use. An online search was performed in the fall of 2021 for all Accreditation Council of Graduate Medical Education (ACGME) approved OBGYN residency programs after initial residency recruitment to capture maximal engagement. Two individual observers (AC, CG) found programs online by searching for full and abbreviated program names in addition to the search terms “OBGYN”, “obstetrics”, and “gynecology” as previously described in the literature [5, 8, 21]. For every social media account, the number of followers, following, and posts were obtained (October 27, 2021 for Instagram and December 3, 2021 for X) shortly after the submission deadline for residency applications. X posts, or formerly tweets, were not analyzed given total number are not easily available and content differs from Instagram. Instagram stories were not evaluated as this is temporary content for 24 hours and difficult to extract given the number of programs. Duration of social media account existence was abstracted from the date of the program’s first post on Instagram while X publicly posts this data. Program rank was determined using the Doximity residency navigator tool by reputation in 2022 [22]. The FREIDA database was queried to determine the type of residency program: academic, community, or combined. Both FREIDA and Doximity were used to obtain the number of residents in the program. If these were not in congruence, the residency home page was visited, and number of residents counted manually. Doximity was used to determine the location of the program, and city size was extrapolated from the location using the U.S. Census Bureau’s statistical area [23]. Populations were defined as large metropolitan (1.5 million or more people), metropolitan (500,000 to 1.5 million people), medium-size urban (200,000–500,000 people), and small urban (50,000–200,000 people). Inquiries were also sent to each program on Instagram by direct message by author CG to ask what type of program they identify as and who posts the content on their Instagram account. Informed consent to participate was obtained with a written response. If their program type direct message was incongruent with FREIDA, FREIDA was utilized for program type given this is a nationwide database. Reply time from direct messenger was recorded. The home institution of this study was excluded from this part of the analysis. Instagram program pages were also searched to identify if they had a highlight reel and if their biography indicated who posted content. Programs were recorded as having a diversity post if there was at least one post primarily regarding any of the following: gender, social and ethnic background, or sexual orientation.

The thirty OBGYN programs with the most Instagram followers were identified. Their last thirty Instagram posts on and prior to October 27, 2021 were analyzed independently by two reviewers (AC, CG). The classification of these posts was adapted from Azoury et al and Abbas et al and changed to reflect specialty differences in content [5, 24]. Posts were categorized into educational, informational, awards/Match, social, wellness, surgical, class, research, advocacy, diversity, and other (S1 Fig). Additionally, the top thirty program’s Instagram highlight reels were categorized by the same two reviewers. Separate classifications were given to these posts as they include video content: educational, research, informational, social, wellness, advocacy/diversity, rotations, location, biography, question & answer (Q&A), day in the life (DITL), and other (S2 Fig). Disagreements between the reviewers were resolved by consensus.

Summary statistics were provided for the characteristics of programs with an Instagram and/or X presence. Mean and standard deviation were presented for continuous variables and frequency and percentage for categorical variables. A multivariate linear regression model was used to evaluate the association of factors with the number of followers on both Instagram and X. A linear mixed model was used to evaluate the association of post content with the number of likes on Instagram with random intercept for the correlation of the repeated measures within the program. The linear mixed model also adjusted for the program size, city size, and type of program. False discovery rate was used for the adjustment of multiple comparisons and control for a possible false positive rate in the linear mixed model. All tests were two-sided and assessed for significance at the 5% level using SAS v9.4 (SAS Institute, Cary, NC).

## Results

Of the ACGME accredited OBGYN residency programs, 88.5% (N = 262/296) programs were identified to have an Instagram account. Of the 34 that did not have an Instagram, 11 programs had just received their initial accreditation in 2021 and 7 were military programs. Fewer programs, 33.7% (N = 97/296), were found to have an X account. Notably, 28.8% (N = 28/97) X accounts and 1.5% (N = 4/262) of Instagram accounts were departmental and not specific to the OBGYN residency program. Most programs created an Instagram (60.3%, N = 158/262) or X (53.6%, N = 52/97) account in 2020.

On Instagram, baseline characteristics were collected showing most programs had an average of 821.2 posts (Standard Deviation/SD = 370.5), were in large metropolitan areas (59.9%), classified themselves as academic programs (46.6%), and had a highlight reel (88.2%). Approximately 60% of programs had at least one post about diversity in any of their posts on Instagram. Most programs were run by residents (84.4%, N = 108/128). Of the 37% (N = 97) programs that responded, thirty-one percent of respondents identified their program as a different type from that listed in the FREIDA database (Table 1).

**Table 1:** Baseline program characteristics.

Category	Instagram (N = 262)	X (Twitter) (N = 97)
**% of all ACGME Programs**	88.5%	33.7%
F**ollowers** (Mean, SD)	821.2 +/- 370.5	467.0 +/- 549.3
**Following** (Mean, SD)	251.9 +/- 195.1	155.4 +/- 195.6
**Doximity Ranking** (Mean, SD)	138.9 +/- 83.0	111.0 +/- 81.4
**Duration** (Mean months, SD)	21.7 +/- 12.9	34.1 +/- 33.6
**Program Size** (Mean, SD)	21.9 +/- 9.2	24.9 +/- 9.0
**Posts** (Mean, SD)	114.8 +/- 108.0	NA
**Posts per Month** (Mean, SD)	5.8 +/- 5.5	NA
**City Size** (%, N)		
Large Metropolitan	59.9% (157)	65.0% (63)
Metropolitan	23.7% (62)	24.7% (24)
Medium-size Urban	11.5% (30)	7.2% (7)
Small Urban	4.9% (13)	3.1% (3)
**Program Type** (%, N)		
Community	16.0% (42)	9.3% (9)
Academic	46.6% (122)	67.0% (65)
Combined	37.4% (98)	23.7% (23)
**Highlight Reel** (% Yes, N)	88.2% (231)	NA
**Diversity Post** (% Yes, N)	59.5% (156)	NA
**Who runs Instagram** (%, N = 128)		
Residents Alone	84.4% (108)	NA
Program Director/Staff	3.9% (5)	NA
Resident + Other Staff	7.8% (10)	NA
Other Admin Staff	3.9% (5)	NA
**Direct Message Response**		
Total Programs (%, N)	37% (97)	NA
Time (Mean days, SD)	12.0 (11.1)	NA
Incongruent program type (%, N)	30.9% (30)	NA

For Instagram and X, programs were evaluated for followers, city size, program type, etc. Instagram also was evaluated for unique characteristics including highlight reels.

*NA, not applicable

On Instagram, many factors were associated with increased number of followers (Table 2). The number of followers increased with more residents, specifically 5.38 more followers per resident (Standard Error/SE = 1.64, p = 0.001). With one more month on Instagram, programs had 4.88 (SE = 1.34, p<0.001) more followers. Programs that followed more individuals had more followers (β estimate = 0.53, SE = 0.06, p<0.001). More posts were associated with more followers (β = 1.00, SE = 0.17, p<0.001). The frequency of posts, or number of posts per month, was not associated with increased followers (p = 0.721). More followers were observed in academic than community (β = 109.2, SE = 39.22, p = 0.006) or combined programs (β = 125.87, SE = 33.63, p = 0.001). The programs in the top quartile (or top 25%) on Doximity had more followers than those in the lower rankings (p = 0.001). The programs with a highlight reel (β = 146.46, SE = 36.56, p<0.001) or diversity post (β = 87.30, SE = 27.85, p = 0.002) had more followers.

**Table 2:** Factors associated with followers on Instagram and X.

	Instagram		X (Twitter)	
Factor	Difference in Followers (SE)	P value	Difference in Followers (SE)	P value
**Program Size**	5.38 (1.64)	**0.001**	2.52 (4.86)	0.606
**Duration in months**	4.88 (1.34)	**<0.001**	5.31 (0.99)	**<0.001**
**# Following**	0.53 (0.06)	**<0.001**	1.55 (0.18)	**<0.001**
**# Posts**	1.00 (0.17)	**<0.001**	NA	NA
**# Posts per Month**	-0.92 (2.58)	0.721	NA	NA
**City Size**	-	0.587	-	0.587
Small Urban ^a^	-	-	-	-
Large Metropolitan	30.73 (54.74)	0.575	-109.80 (115.31)	0.344
Metropolitan	16.81 (56.45)	0.766	-118.71 (120.04)	0.326
Medium-size Urban	-22.34 (60.38)	0.712	-	-
**Program Type**	-	<**0.001**	-	0.394
Community ^a^	-	-	-	-
Academic	109.20 (39.22)	**0.006**	19.50 (136.29)	0.887
Combined	-16.72 (35.14)	0.635	-90.14 (132.16)	0.497
**Doximity Ranking**	-	**0.002**	-	**0.012**
Top Quartile (≥ 25%) ^a^	-	-	-	-
Second Quartile (25%-50%)	-109.87 (33.63)	**0.001**	-174.29 (92.69)	0.063
Third Quartile (50%-75%)	-120.21 (36.71)	**0.001**	-329.43 (97.12)	**0.001**
Low Quartile (≥ 75%)	-140.49 (40.93)	**0.001**	-275.06 (123.55)	**0.029**
**Highlight Reel**	-	-		
No ^a^	-	-		
Yes	146.46 (36.56)	**<0.001**		
**Diversity Post**	-	-		
No ^a^	-	-		
Yes	87.30 (27.85)	**0.002**		

Factors including program size, city size, program type, and Doximity ranking were evaluated to identify any association with followers on Instagram and X. A multilinear regression model was used to calculate the beta estimate, or difference in followers per factor. Significant p values are bolded.

^a^ Reference

*NA, not applicable

On X, the average number of followers was 467.0 (+/- 549.3) and duration on the platform was 34.1 months (+/- 33.6). Most programs were in large metropolitan areas (65.0%) and classified themselves as academic programs (67.0%) (Table 1). The number of followers was significantly associated with duration in months on X since the first post, number following, and Doximity ranking (Table 2). On average, programs had 5.31 more followers per month on X (SE = 0.99, p<0.001). As programs followed other social media users on X, they gained more followers (β = 1.55, SE = 0.18, p<0.001). The program in the top quartile on Doximity ranking had more followers than those in the lowest 50% of rankings (p = 0.029).

**Fig 1:**
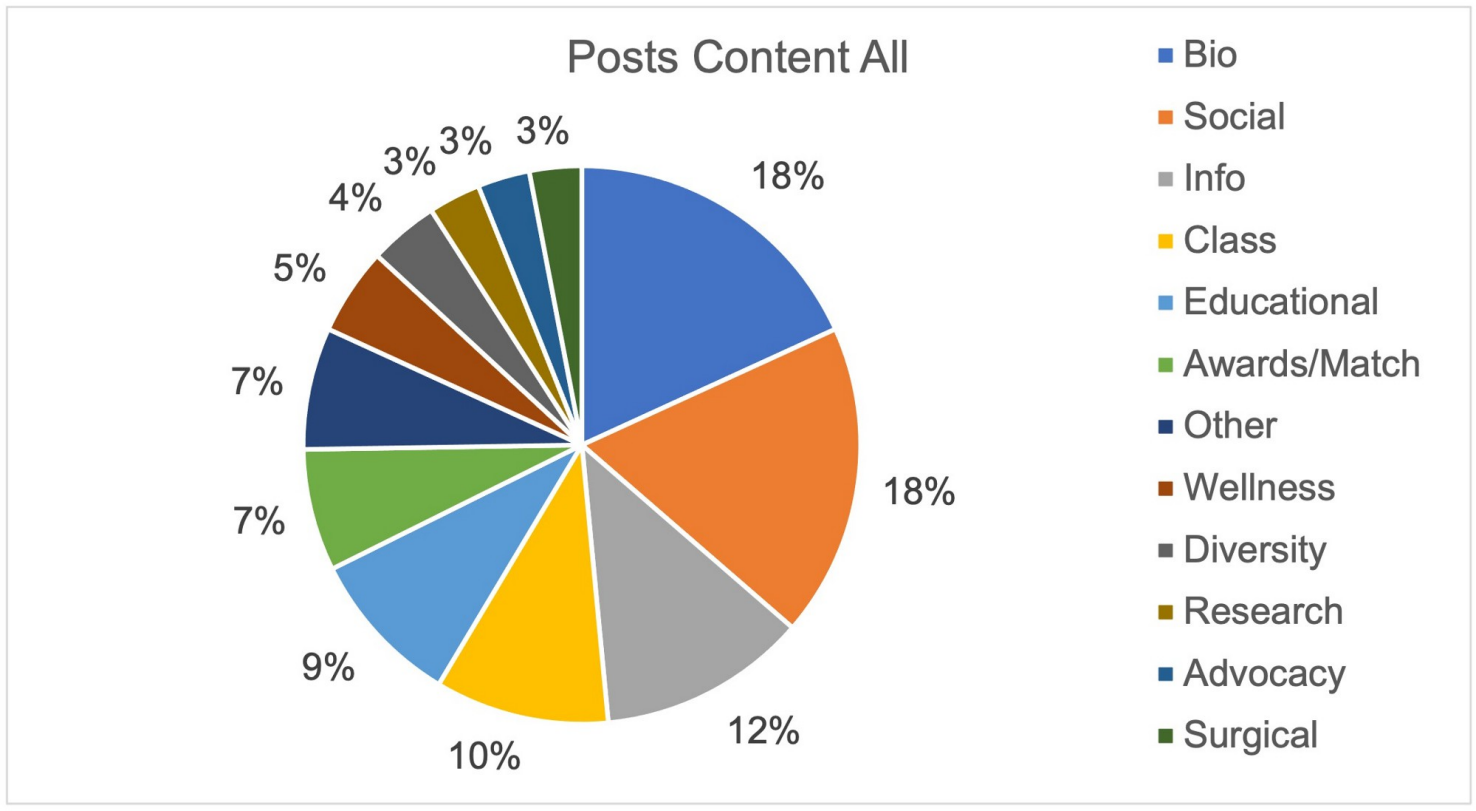
Instagram post content, all. Distribution of content for the last thirty posts for the top thirty OBGYN programs.

**Fig 2:**
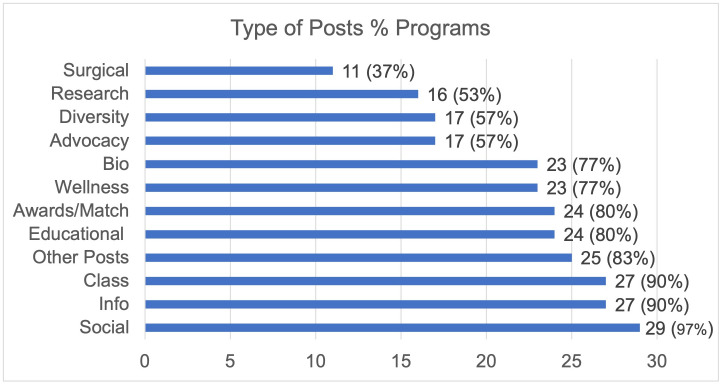
Instagram post content, top 30. Distribution on the % of the top thirty OBGYN programs that posted that specific type of content in the last thirty posts.

**Fig 3:**
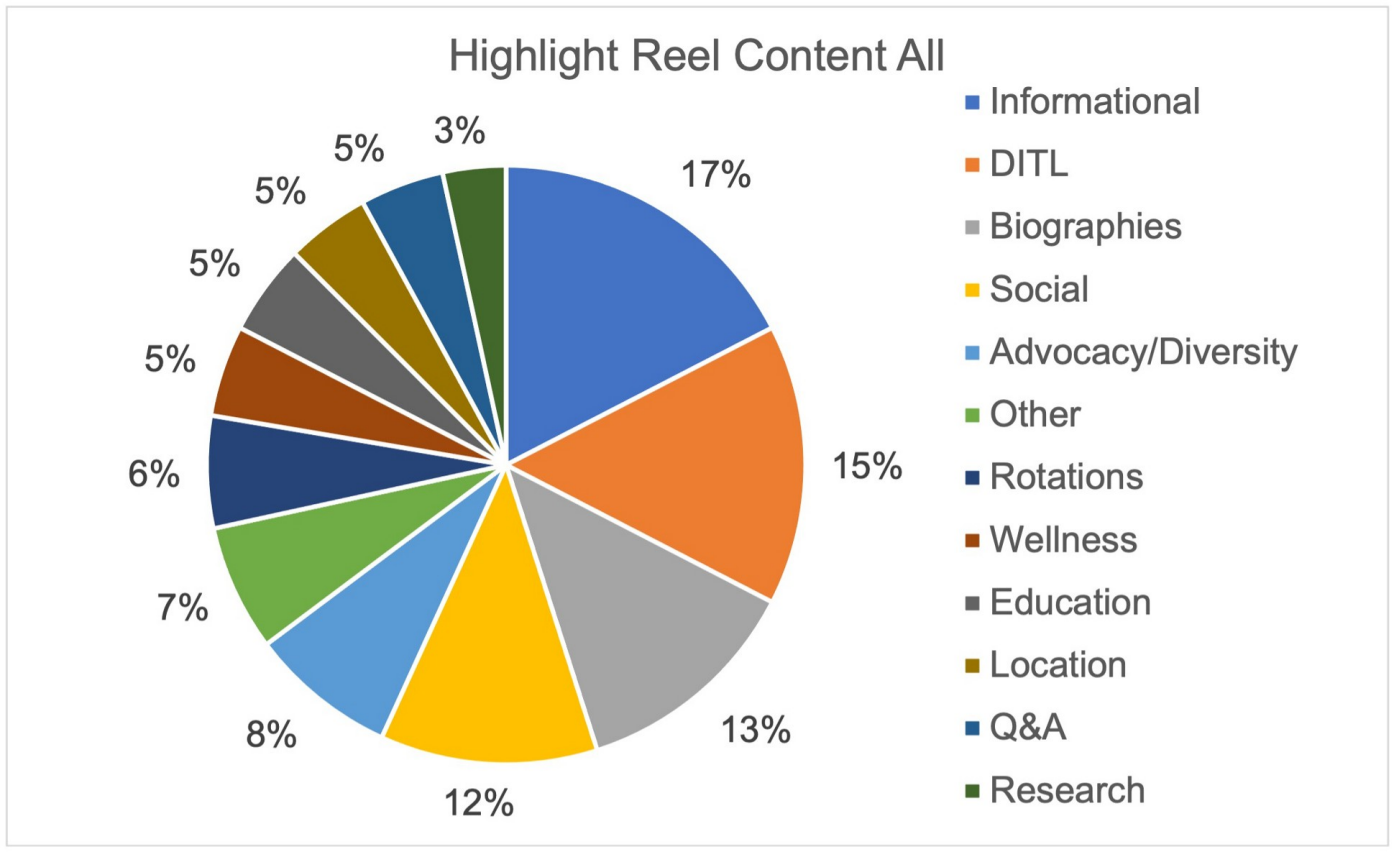
Instagram highlight reel content, all. Distribution of content for all posted highlight reels for the top thirty OBGYN programs.

**Fig 4:**
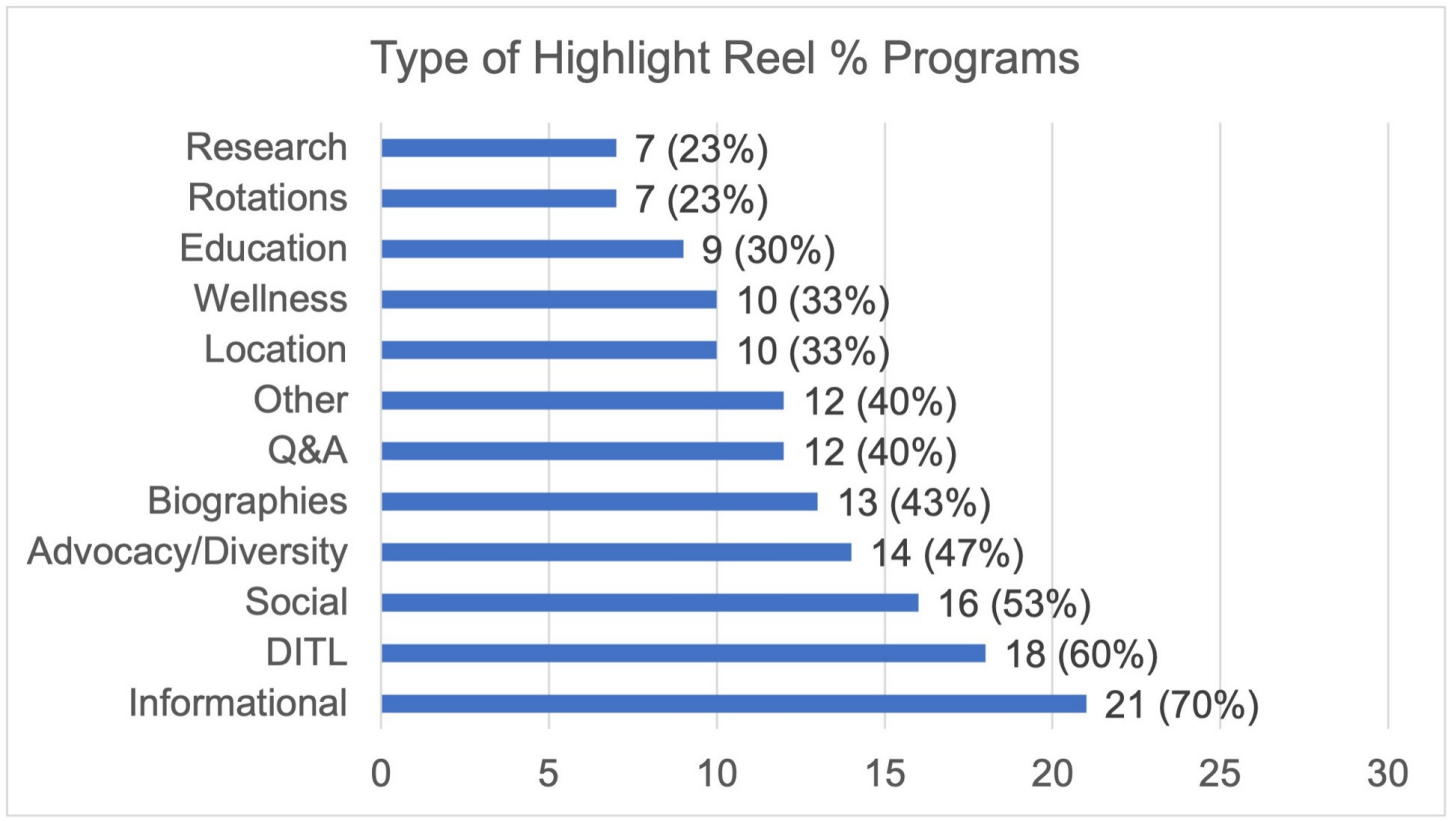
Instagram highlight reel content, top 30. Distribution of the % of the top thirty OBGYN programs that posted that specific type of content in their highlight reels.

An analysis of 900 posts from the top thirty most followed programs on Instagram demonstrated most of the content was biographical (18%) or social in nature (18%) (Figs 1 and 2). The least posted content overall was surgical (3%), research (3%), and advocacy (3%). In the top thirty programs, social content (97%) followed by information (90%) and class (90%) were posted at least once in their last thirty posts while surgical was the least common with only 37% of programs having a post related to surgery. Highlight reels for the top thirty programs were evaluated (Figs 3 and 4). One program had no highlight reels. Most of the content was information (17%) or related to DITL (15%). The least posted content overall was research (3%), Q&A (5%), and location (5%). Of the top thirty programs, informational content (70%) followed by DITL (60%) and social (53%) were the most common content posted by programs by having at least one highlight regarding this content while research, and rotations were least common with only 23% of programs having a highlight reel dedicated to these.

**Fig 5:**
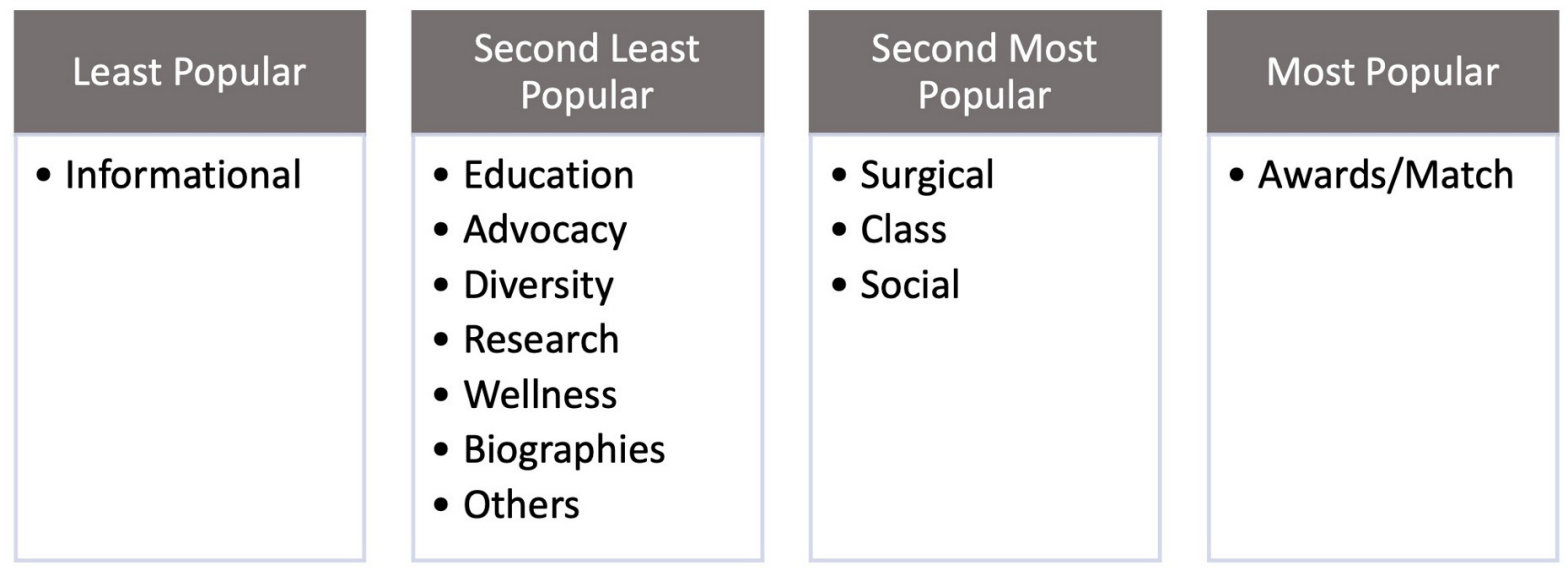
Instagram post content popularity. Based off the linear mixed model of content likes in A.4, content tiers were created based on significant and insignificant p values between content types showing the most to least popular Instagram post content.

Type of Instagram post content was significantly associated with the number of likes after controlling for other characteristics such as program size, program type, and city size (p<0.001) (S1 Table). Next, content was compared to one another to identify superiority after the false discovery rate adjustment for multiple comparisons between content. Advocacy posts had more likes than informational posts (p = 0.007). More likes were observed for awards/Match posts than advocacy, biography, class, diversity, informational, surgical, others, research, social, wellness, and educational (p<0.001). Biography posts had more likes than informational but less than class, and social posts (p≤0.007). More likes were detected for class posts than diversity, informational, research, and educational (p≤0.008). Social posts had more likes than diversity or educational posts (p<0.005). Less likes were seen for informational post than surgical, others, social, and wellness (p≤0.004) (S2 Table). Overall, posts about awards and/or Match received the most likes while informational received the least (Fig 5).

## Discussion

Our study shows that most OBGYN residency programs (88.5%) have adopted Instagram while only 33.7% of programs adopted X. Most of these programs adopted social media during the COVID pandemic in 2020 as previously demonstrated [13]. Instagram as a social platform may also be more popular amongst many residency specialties given increased usage of Instagram by applicants as seen in other specialties and the ability to more easily engage with photo and video content [8, 25].

This is the first study to establish that the number of followers, or popularity, on Instagram is positively correlated with larger residency program size and academic program type. Yadav et al is the only other published OBGYN study to evaluate popularity on Instagram and calculated Doximity ranking in relation to number of posts, followers, and likes on a program’s last three posts [13]. Similar to our study, Doximity ranking of OBGYN programs was associated with a higher number of followers. However, Yadav et al did not evaluate content or followers in relation to other program traits [13].

Our study sought to further explore modifiable social media factors that can be implemented by programs to attempt to increase followers as previously mentioned factors are likely related to reputation and accessibility to a greater population of users. Modifiable factors that are positively correlated with followers include number of posts for Instagram, following more accounts, and time on each platform, which is consistent with orthopedic and plastic surgery literature [5, 24].

Surprisingly, increased frequency of Instagram posts was not associated with more followers as expected given suspected mutual engagement. This is similar to plastic surgery literature showing only a weak correlation between number of posts and engagement [11]. Likely, there is an optimal post frequency as seen in prior social media data [26]. Further studies are needed to see if this changes over time.

Lastly, biographical and social were the two most posted content types, also consistent with plastic surgery and orthopedic literature [24, 25, 27]. Additionally, awards and/or the Match followed by social, class, and surgical were the most engaged content. Although not all categories were the same, our results are consistent with plastic surgery literature with social posts and accolades (like awards/match) being of high importance [11, 28]. The emphasis on social content may be a consequence of prohibited in-person interaction because of the COVID pandemic and may allow applicants to determine program ‘fit’.

It is important to also highlight the least popular or engaged content includes advocacy, research, diversity, and wellness (Figs 1, 2 and 5). These items typically showcase unique program aspects that may distinguish programs from one another for applicants, but, unfortunately, are the least emphasized. Diversity, equity, and inclusion (DEI) has become integral in promoting and celebrating an environment of diversity for residents, faculty, staff, and patients which is promoted by ACOG DEI Excellence Workgroup [29]. Despite this, 40% of all OBGYN programs did not have one Instagram post about diversity. This finding may reflect resident run accounts emphasizing a social atmosphere rather than the structured content one may expect from a content manager or faculty. In plastic surgery literature, it was shown that posts with a greater average Fitzpatrick skin type had a greater number of likes, the opposite of our study [11]. However, as the authors point out, this is a subjective measure and did not evaluate diversity in its entirety. Our study sought to identify posts including many forms of diversity. Additionally, plastic surgery literature is congruent in our results showing wellness is in the minority posted, despite the recent emphasis on combating burnout in medicine [30]. Ultimately, programs should consider including content that represents their program’s unique aspects and core values.

Post accuracy also should be of great importance to programs. A negative impact on applicant’s perception of a residency program in both orthopedic and plastic surgery literature has been secondary to a program’s social media [8, 25]. Specifically, 11% of plastic surgery applicants never trusted a program’s social media information or posts [8]. In our study, 31% of social media accounts reported a different program type than that listed on FREIDA. This either reflects an inaccuracy in the FREIDA database or those who post the content, 90% of which were resident run, among programs who responded. While this may reflect a poorly updated FREIDA database, it is important to ensure that there is accuracy in what is being posted online, especially if programs are placing this burden on residents.

Unfortunately, social media training is variable and professional guidelines are lacking [31]. The American College of Surgeons released a social media statement including a review of 7 national and international organizations, most notably American Medical Association (AMA) and ACOG, which highlights professional web page content and appropriate communication between physicians, patients, and colleagues on social media but not in regards to residency recruitment [32–34]. Neither AMA nor National Resident Matching Program (NRMP) have defined appropriate social media interactions between applicants and programs. Despite this, social media has been used to vet residency applicants. In one study on focused on general surgery, 18% of program directors reported screening applicants through social media. Furthermore, 11% of program directors lowered or removed an applicant from their rank list due to unprofessional content [35]. In another study, at least 15% of plastic surgery applicant respondents were concerned that engaging with a program’s social media would attract attention to their own [8]. Overt bias and judgement related to social media content posted by trainees personal accounts can even be found in recent academic literature [36].

A council of residency directors in Emergency Medicine was the first and only specialty to create social media guidelines in 2014 [37]. These guidelines recommend content should be designated to a content manager, not a trainee, to ensure professional communications and accurate content as violations can interfere with privacy, patient confidentiality, and impartial recruitment, and to employ a uniform policy to screen applicants, if performed, to decrease bias [37]. Program directors should heavily consider whether screening applicants’ social media is beneficial to recruitment and if residents should bear the responsibility of posting social media content [37]. The authors believe all specialties should consider adopting social media guidelines.

Our study is the first OBGYN study to evaluate residency posted content in relation to likes to assess what content is the most engaged instead of content to solely followers. It also uniquely examines highlight reels and diversity posts, both of which were associated with more followers.

Our study has limitations. First, there was no way to distinguish a follower’s background or which users were liking content. Thus, our findings, while reflective of general popularity and engagement, do not necessarily reflect residency applicants’ interactions with social media. Further, some applicants may be afraid of having their own accounts discovered and change their social media profiles during recruitment season [8]. Second, we were only able to ascertain who runs half of the total social media accounts identified either by direct message or as identified in their profile (N = 128/262). Therefore, although a majority were identified as resident run, this may not reflect all OBGYN accounts.

## Conclusions

Understanding what social media content attracts more followers and increases engagement is crucial as resident recruitment may be impacted by content posted by OBGYN programs. Programs should consider following more profiles and posting social and awards and/or the Match content as this may increase followers and engagement. Our findings highlight the need for social media content that accurately reflects residency and departmental mission statements, is pertinent to what applicants are seeking from a program and maintains professionalism. Ultimately, national bodies and residency programs should consider establishing professional social media guidelines for the protection of both residents and applicants.

## Supporting information

S1 Fig Definitions of post content. (DOCX)

S2 Fig Definitions of highlight reel content. (DOCX)

S1 Table Factors associated with likes on Instagram. Factors including program size, city size, program type, and content were evaluated to identify any association of likes for posts on Instagram. A multilinear regression model was used to calculate the beta estimate, or difference in likes per factor.
(DOCX)

S2 Table Content linear mixed model. False discovery rate (FDR) model showing multivariate comparisons between content with only significant associations included in the following table.
(DOCX)

S1 Data (XLSX)

## Abbreviations

DEI: diversity, equity, and inclusion
DITL: day in the life
Q&A: question & answer
