# Supplementary material for: Social media trends in obstetrics and gynecology residency programs on Instagram and X (Twitter)
Source: PLoS One. 2024 May 6;19(5):e0296930. doi: 10.1371/journal.pone.0296930 (PMC11073692; doi:10.1371/journal.pone.0296930)
Supplement: S1 Fig — (DOCX) [file pone.0296930.s001.docx]

**S1 Fig**

| **Educational** | Posts trying to teach a concept, announcing seminars, learning opportunities, podcasts, or presenting physical examination findings and demonstrations |
| --- | --- |
| **Social** | Posts regarding vacations, hobbies, camaraderie, food, sporting events, concerts, or celebration of life events. |
| **Wellness** | Posts regarding wellness activities including posts with #wellness. |
| **Informational** | Posts providing information or promotions about the residency program including ACOG Residency Showcase |
| **Awards/Match** | Posts regarding medical awards and/or The Match, including residency and fellowship match. |
| **Biographies** | Posts regarding current resident or physician biographies. |
| **Class** | Posts highlighting a class of residents or fellows. |
| **Advocacy** | Any post making mention of advocacy for patients and volunteerism. |
| **Research** | Posts regarding publications, abstracts, posters, conferences, research awards, or grants |
| **Surgery** | Posts of images in the OR or promoting operating room experiences. |
| **Diversity** | Posts showcasing diversity in program including race/ethnicity, LGBTQIA, gender, etc. |
| **Other** | Posts not previously mentioned |
