# Supplementary material for: Social media trends in obstetrics and gynecology residency programs on Instagram and X (Twitter)
Source: PLoS One. 2024 May 6;19(5):e0296930. doi: 10.1371/journal.pone.0296930 (PMC11073692; doi:10.1371/journal.pone.0296930)
Supplement: S2 Fig — (DOCX) [file pone.0296930.s002.docx]

**S2 Fig**

| **Location** | Posts regarding information the city the program is in including pictures, activities in the surrounding areas. |
| --- | --- |
| **DITL** | Posts regarding a “Day in the Life” or “Takeover” where an individual posts activities occurring throughout the day. |
| **Advocacy/Diversity** | Anything regarding advocacy or diversity. |
| **Q&A** | Posts regarding question-and-answer sessions or posts. |
| **Biographies** | Posts regarding information specific to the background of classes, faculty, or residents. |
| **Rotations** | Posts regarding specific rotations or resident schedule. |
| **Informational** | Posts regarding information for applicants including ACOG resident week and Showcase, or facts about the program in general. |
| **Wellness** | Posts regarding wellness activities including posts with #wellness. |
| **Education** | Posts trying to teach a concept, announcing seminars, learning opportunities, podcasts, or presenting physical examination findings and demonstrations. |
| **Social** | Posts regarding vacations, hobbies, camaraderie, food, sporting events, concerts, or celebration of life events. |
| **Other** | Posts not previously mentioned. |
