# Supplementary material for: Social media trends in obstetrics and gynecology residency programs on Instagram and X (Twitter)
Source: PLoS One. 2024 May 6;19(5):e0296930. doi: 10.1371/journal.pone.0296930 (PMC11073692; doi:10.1371/journal.pone.0296930)
Supplement: S1 Table — Factors including program size, city size, program type, and content were evaluated to identify any association of likes for posts on Instagram. A multilinear regression model was used to calculate the beta estimate, or difference in likes per factor. (DOCX) [file pone.0296930.s003.docx]

**S1 Table**

| **Factor** | **Difference in Likes (SE)** | **P value** |
| --- | --- | --- |
| **Program Size** | 0.84 (0.47) | 0.074 |
| **City Size** | - | 0.590 |
| Medium-size Urban (ref) | - | - |
| Large Metropolitan | -18.36 (21.77) | 0.399 |
| Metropolitan | -24.06 (23.52) | 0.307 |
| **Program Type** | - | 0.379 |
| Community (ref) | - | - |
| Academic | 22.23 (22.29) | 0.319 |
| Combined | 36.59 (26.57) | 0.169 |
| **Content** | - | **<0.001** |
| Educational (ref) | - | - |
| Advocacy | 14.74 (9.18) | 0.189 |
| Awards/Match | 83.06 (7.14) | **<0.001** |
| Bio | 4.64 (6.20) | 0.455 |
| Class | 29.89 (6.68) | **<0.001** |
| Diversity | -2.82 (8.66) | 0.745 |
| Info | -12.38 (6.54) | 0.059 |
| Surgical | 23.43 (10.01) | **0.020** |
| Others | 15.55 (7.18) | **0.031** |
| Research | 3.69 (9.04) | 0.683 |
| Social | 21.95 (6.05) | **<0.001** |
| Wellness | 13.09 (8.17) | 0.110 |
