# Supplementary material for: Social media trends in obstetrics and gynecology residency programs on Instagram and X (Twitter)
Source: PLoS One. 2024 May 6;19(5):e0296930. doi: 10.1371/journal.pone.0296930 (PMC11073692; doi:10.1371/journal.pone.0296930)
Supplement: S2 Table — False discovery rate (FDR) model showing multivariate comparisons between content with only significant associations included in the following table. (DOCX) [file pone.0296930.s004.docx]

**S2 Table**

| **Content** | **Reference** | **Difference**  **in Likes** | **SE** | **P value** | **FDR**  **Adjusted P** |
| --- | --- | --- | --- | --- | --- |
| Advocacy | Awards/Match | -68.33 | 9.26 | <.0001 | <.0001 |
| Advocacy | Info | 27.11 | 8.87 | 0.0023 | 0.0069 |
| Awards/Match | Bio | 78.43 | 6.34 | <.0001 | <.0001 |
| Awards/Match | Class | 53.18 | 6.68 | <.0001 | <.0001 |
| Awards/Match | Diversity | 85.88 | 8.67 | <.0001 | <.0001 |
| Awards/Match | Info | 95.44 | 6.63 | <.0001 | <.0001 |
| Awards/Match | OR/Surgical | 59.63 | 10.17 | <.0001 | <.0001 |
| Awards/Match | Other Posts | 67.51 | 7.24 | <.0001 | <.0001 |
| Awards/Match | Research | 79.37 | 9.08 | <.0001 | <.0001 |
| Awards/Match | Social | 61.12 | 6.20 | <.0001 | <.0001 |
| Awards/Match | Wellness | 69.98 | 8.230 | <.0001 | <.0001 |
| Awards/Match | Educational | 83.06 | 7.14 | <.0001 | <.0001 |
| Bio | Class | -25.25 | 5.65 | <.0001 | <.0001 |
| Bio | Info | 17.01 | 5.54 | 0.0022 | 0.0069 |
| Bio | Social | -17.31 | 5.09 | 0.0007 | 0.0025 |
| Class | Diversity | 32.70 | 8.20 | <.0001 | 0.0003 |
| Class | Info | 42.26 | 6.03 | <.0001 | <.0001 |
| Class | Research | 26.20 | 8.74 | 0.0028 | 0.0081 |
| Class | Educational | 29.89 | 6.68 | <.0001 | <.0001 |
| Diversity | Social | -24.76 | 7.81 | 0.0016 | 0.0052 |
| Info | OR/Surgical | -35.81 | 9.67 | 0.0002 | 0.0009 |
| Info | Other Posts | -27.93 | 6.63 | <.0001 | 0.0001 |
| Info | Social | -34.32 | 5.44 | <.0001 | <.0001 |
| Info | Wellness | -25.46 | 7.73 | 0.0010 | 0.0035 |
| Social | Educational | 21.95 | 6.05 | 0.0003 | 0.0012 |
